# Enteric disease surveillance under the AFHSC-GEIS: Current efforts, landscape analysis and vision forward

**DOI:** 10.1186/1471-2458-11-S2-S7

**Published:** 2011-03-04

**Authors:** Nisha N Money, Ryan C Maves, Peter Sebeny, Matthew R Kasper, Mark S Riddle, the AFHSC-GEIS Enteric Surveillance Writing Group

**Affiliations:** 1 Armed Forces Health Surveillance Center, 503 Robert Grant Avenue, Silver Spring, MD 20910, USA; 2 Naval Medical Research Center Detachment, Centro Medico Naval “CMST,” Av. Venezuela CDRA 36, Callao 2, Lima, Peru; 3 Naval Medical Research Unit No. 3, Extension of Ramses Street, Adjacent to Abbassia Fever Hospital, Postal Code 11517, Cairo, Egypt; 4 Naval Medical Research Unit Number 2, Kompleks Pergudangan DEPKES R.I., JI. Percetakan Negara II No. 23, Jakarta 10560, Indonesia; 5 Naval Medical Research Center, 503 Robert Grant Avenue, Silver Spring, MD 20910, USA; 6 US Army Medical Research Unit Kenya (USAMRU-K), U.S. Embassy, Attention: MRU, United Nations Avenue, Post Office Box 606, Village Market 00621 Nairobi, Kenya; 7 U.S. Navy Environmental Preventive Medicine Unit No. 2, 1887 Powhatan Street, Norfolk, VA 23511, USA

## Abstract

The mission of the Armed Forces Health Surveillance Center, Division of Global Emerging Infections Surveillance and Response System (AFHSC-GEIS) is to support global public health and to counter infectious disease threats to the United States Armed Forces, including newly identified agents or those increasing in incidence. Enteric diseases are a growing threat to U.S. forces, which must be ready to deploy to austere environments where the risk of exposure to enteropathogens may be significant and where routine prevention efforts may be impractical. In this report, the authors review the recent activities of AFHSC-GEIS partner laboratories in regards to enteric disease surveillance, prevention and response. Each partner identified recent accomplishments, including support for regional networks. AFHSC/GEIS partners also completed a Strengths, Weaknesses, Opportunities and Threats (SWOT) survey as part of a landscape analysis of global enteric surveillance efforts. The current strengths of this network include excellent laboratory infrastructure, equipment and personnel that provide the opportunity for high-quality epidemiological studies and test platforms for point-of-care diagnostics. Weaknesses include inconsistent guidance and a splintered reporting system that hampers the comparison of data across regions or longitudinally. The newly chartered Enterics Surveillance Steering Committee (ESSC) is intended to provide clear mission guidance, a structured project review process, and central data management and analysis in support of rationally directed enteric disease surveillance efforts.

## Background

Enteric infections pose a significant risk to the 80 to 100 million travelers from industrialized countries visiting developing countries [1] and are leading causes of death among children in these same developing countries, where they claimed between 1.4 and 2.5 million lives in the year 2000 [2]. From the perspective of the United States Department of Defense (DoD), political instabilities in many parts of the world require that U.S. military personnel must be ready to deploy to austere environments where the risk of exposure to infectious diseases may be significant and where routine preventive health efforts are often impractical. Acute diarrheal illness has played a significant role in the outcomes of military campaigns throughout history [3-5]. Advances in environmental health interventions and effective therapies have been insufficient to eliminate the burden of enteric infections in deployed military personnel, which can result in lost work days, increased health care utilization, and compromised operational readiness and effectiveness [5-9].

For these reasons, enteric disease surveillance was established as a pillar within the AFHSC-GEIS system. This system is intended to unite the resources of DoD research facilities and the military health system to facilitate the rapid recognition and understanding of, and response to, infectious disease threats to protect global health and that of the U.S. forces. Herein, the authors report on past and current accomplishments of enteric surveillance efforts within AFHSC-GEIS, provide an assessment of how these efforts fit within a changing landscape of global enteric disease research efforts, and discuss future efforts to improve coordinated efforts within the program.

## Current AFHSC-GEIS enteric surveillance efforts

AFHSC-GEIS currently supports enteric surveillance activities at the five overseas DoD research laboratories and at the Naval Environmental and Preventive Medicine Unit Two (NEPMU-2) in Norfolk, Va. The reach of these laboratories extends beyond their respective host countries, as each institution has its own network of regional activities, working with neighboring countries as well as their primary host. Table 1 lists some of the accomplishments of the laboratories participating in the AFHSC-GEIS network, including training host nation personnel, providing reference laboratory capabilities and investigating outbreaks. The following vignettes describe representative efforts within this surveillance network, selected based on their diversity and impact.

**Table 1:** Recent Accomplishments of DoD Laboratory Partners in Enteric Disease Surveillance Under AFHSC-GEIS

Partner	Clinical Surveillance and Capacity Building	Laboratory Results/Accomplishments
AFRIMS	• Pediatric case-control study • Enteric surveillance in troops deploying to Cobra Gold 2009 (Thailand) and Balaktan 2009 (Philippines) • Defining etiology of AGE and antimicrobial resistance among indigenous populations in South Asia and Southeast Asia	• Sites in Nepal and Thailand have enrolled more than 2500 cases and controls during the past year • High rates of fluroquinolone and TMP-SMX resistance among pediatric and travel-associated *Campylobacter* isolates. • Most *Shigella* species are TMP-SMX resistant

NAMRU-3	• Birth cohort research and epidemiology • Severe diarrhea study at Cairo University • Case-control study of modifiable risk behaviors • Molecular biology and cholera/rotavirus microbiology reference center for the Middle East and Africa • Training workshops and courses for laboratorians from Afghanistan, Djibouti, Ghana, Iraq, Jordan, Libya, Morocco, Sudan • Biennial enteric disease surveillance in Operation Bright Star (Egypt)	• 2223 children enrolled to describe pathogen distribution (2000 to 2005) • 117 cases and 1:1 age-matched controls assessed for risk behaviors including food and water sources • 303 *V. cholerae* isolates archived and characterized—2 serotypes with widespread antimicrobial resistance • 937 stool samples processed from eight countries to date this year in capacity as WHO Rotavirus Reference Laboratory • Norovirus outbreak response support, Incirlik Air Base, Turkey

NMRCD-Peru	• Cohort study among basic combat trainees • Antimicrobial surveillance testing in Lima and five departments of Peru	• Received 2159 specimens for antimicrobial surveillance and confirmed bacterial pathogens in 83 percent of them. • The cohort study enrolled 381 participants with 84 diarrheal cases with bacterial pathogens confirmed in 42 (50 percent).

NAMRU-2	• 12,000 specimens from Indonesian pediatric diarrhea • Surveillance project covered six cities on five islands and identified rotavirus as the leading causative agent	• Advanced characterization of *Campylobacter* spp. and *Shigella* spp.

USAMRU-K	• Movement of Enteric Microbiology Laboratory from Nairobi to Kericho includes all ages case-control protocol at Kericho District Hospital and two additional district hospitals in Kisumu	• Detected and identified bacterial pathogens in 28 percent of diarrheal stool specimens • Renovated infrastructure to enable facility relocation to the new Microbiology Hub in Kericho (MHK) • Five-year surveillance protocol uses case-control approach, broadens testing spectrum, and includes antibiotic susceptibility testing • Clinical and (FDA-approved) molecular epidemiology of diarrheal illnesses

NEPMU-2	• Establishing diagnostic capability for Norovirus VGE collection kits with thermal shipping boxes deployed to 30 ships	• Expected to start processing kits in early 2010

Acronyms: Acute gastroenteritis (AGE), Food and Drug Administration (FDA), trimethoprim-sulfamethoxazole (TMP-SMX), viral gastroenteritis (VGE), World Health Organization (WHO)

## Pediatric case-control study—Armed Forces Research Institute of Medical Sciences (AFRIMS), Bangkok, Thailand

AFRIMS was established over 40 years ago as a tropical disease research and development institution within DoD. The Department of Enteric Diseases at AFRIMS has a sustained history of conducting collaborative research, epidemiology, and preclinical and clinical trials on enteric diseases. In addition to supporting enteric surveillance among U.S. troops deploying on military training operations such as Cobra Gold 2009 (Thailand) and Balakatan 2009 (Philippines), AFRIMS, through AFHSC-GEIS support, has continued a project of defining the etiologies of pediatric acute gastroenteritis and antimicrobial resistance patterns among host country populations in South Asia (Nepal) and Southeast Asia (Thailand). Under these surveillance projects, over 2,500 cases and controls were enrolled during the past year. This study has continued to demonstrate the importance of particular bacterial and viral pathogens in these regions and the levels of antimicrobial resistance among the most common bacterial etiologies—providing critical information for treatment consideration in individuals deploying to these regions (e.g., use of azithromycin for empiric therapy among travelers/deployments to Southeast Asia).

## Establishment of a reference laboratory for a regional cholera Network—Naval Medical Research Unit no. three (NAMRU-3), Cairo, Egypt

Over the past decade, NAMRU-3 has served as a core laboratory for enteric disease research and epidemiology in pediatric populations through a series of birth cohorts [10-13] and other surveillance studies and clinical trials conducted among U.S. military populations deployed to Egypt and the surrounding region [6-8,14-24]. This track record in enteric disease surveillance and research, modern laboratory and experienced staff has positioned NAMRU-3 as a hub for enteric surveillance activities in the region. In the past year, AFHSC-GEIS has supported pediatric hospital studies at Cairo University, where 140 participants with severe diarrhea have been enrolled in the last year. NAMRU-3 and AFHSC-GEIS, in collaboration with two Egyptian university medical centers, have also initiated a case-control study looking at modifiable risk factors where a total of 164 cases and controls have been enrolled to date. These projects will provide important data about pathogen distribution, and the epidemiological, clinical, economic and molecular characteristics of diarrhea in Egypt. They will also provide the opportunity to explore novel pathogen discovery.

NAMRU-3 has established a *Vibrio cholerae* microbiology and molecular biology reference center for Africa and the Middle East and serves as a reference laboratory for rotavirus characterization in partnership with the World Health Organization (WHO) in an effort to understand and identify the emergence of novel viral strains under vaccine pressure in the region. Both projects have established links with researchers and governmental organizations in countries affected by cholera and rotavirus, and worked to characterize the isolates while providing training opportunities for the host country’s public health community. To date, this initiative has established 16 active sites, trained over 53 people, and collected and tested 1,257 specimens. The establishment of this reference center activity is an example of how regional laboratories can serve an important global health surveillance function and build capacity and capability in developing countries.

## Enteric disease surveillance among Peruvian Military Recruits—Naval Medical Research Center Detachment (NMRCD), Lima, Peru

NMRCD was established in 1983 as a joint enterprise of the U.S. Navy and the Peruvian Navy with a mission to conduct research on infectious diseases of military and public health importance in Peru and Latin America. AFHSC/GEIS supports enteric disease research at NMRCD through funding of prospective military cohorts, antimicrobial resistance surveillance in enteric pathogens and a passive surveillance network for febrile diseases including diarrhea.

In collaboration with the Peruvian Army, NMRCD has conducted a prospective cohort of diarrheal disease incidence and prevalence among Peruvian soldiers in the Peruvian Amazon. To date, over 2,900 participants have enrolled in the cohort alone. Baseline stool and serum specimens are obtained from participants, and active surveillance is conducted to detect and evaluate cases of acute diarrhea. Fecal specimens are tested by means of conventional culture, by PCR-based methods for the detection of diarrheagenic *Escherichia coli* (DEC), and by microscopy and ELISA for parasitic pathogens.

Ongoing results at Vargas Guerra show an annual incidence of diarrhea from 0.31-0.70 episodes of diarrhea per person-year since the project began in 2003. Prior to the establishment of active surveillance at the study site in 2004, 40 percent of diarrhea cases presenting for medical care were confirmed as *Shigella* (primarily *S. flexneri* 2a and 3a) by culture [25]. With active case finding, an increased number of less-severe cases were identified, with the proportion of shigellosis as a cause of diarrhea decreasing to a lower but still significant 24 percent of cases. *S. flexneri* is a pathogen of major military and public health importance and is the target of vaccine development by the Walter Reed Army Institute of Research (WRAIR) and the Military Infectious Diseases Research Program (MIDRP). NMRCD also conducts antimicrobial drug resistance surveillance for enteric pathogens in Peru with support from AFHSC-GEIS. Last year, NMRCD obtained 2,159 isolates from collaborating project sites in five departments of Peru with differing climates and population densities. From the isolates, 1,802 bacterial pathogens were confirmed. Of these 1,802 specimens, 47.8 percent were confirmed as *Shigella* spp., 5.4 percent as *Salmonella* spp., 35.7 percent as *Campylobacter* spp., and 6.7 percent as diarrheagenic *E. coli* (DEC). Among organisms evaluated, *Campylobacter* spp. showed high rates of resistance to fluoroquinolones (91.6 percent), although local rates of fluoroquinolone resistance were much lower in the Amazon basin (28 percent). Overall rates of macrolide resistance were low for *Campylobacter* spp. (azithromycin 1.9 percent, erythromycin 3.0 percent). *Shigella* strains were broadly resistant to trimethoprim-sulfamethoxazole (88.3 percent), with lower but noteworthy resistance rates to that agent noted in salmonellae (19.4 percent). *Salmonella* spp. was generally resistant to erythromycin (90.8 percent) but susceptible to fluoroquinolones (1.0 percent resistant to ciprofloxacin) and azithromycin (7.1 percent resistant). DEC had broadly preserved quinolone susceptibility, with only one EPEC isolate out of 120 DEC noted to be resistant to ciprofloxacin.

Related enteric projects include the genetic characterization of antimicrobial resistance mechanisms in enteric pathogens from Peruvian children with diarrhea, in collaboration with colleagues at the Universidad Peruana Cayetano Heredia and the Instituto de Investigación Nutricional (both in Lima). Specific activities include the detection of efflux pumps and other fluoroquinolone resistance mechanisms in DEC, and the identification of extended-spectrum beta-lactamases in DEC.

## Advanced characterization of enteric pathogens in Indonesian children—Naval Medical Research Unit no. two (NAMRU-2), Jakarta, Indonesia

NAMRU-2 conducts disease surveillance, research, outbreak response, capacity building and training throughout Southeast Asia, including in Cambodia, Indonesia and the Lao People’s Democratic Republic. NAMRU-2 contributed to the identification of the first case of rotavirus strain G12 in Indonesia, an important finding for emerging pathogen surveillance [26]. The combination of the P[6] genotype in this rotavirus strain leads to the potential of zoonotic transmission and is important for vaccine development and identification of novel and emerging rotavirus strains. In addition, capitalizing on over 12,000 specimens collected from an Indonesian pediatric diarrhea surveillance effort, NAMRU-2 investigators evaluated the molecular epidemiology and antimicrobial resistance patterns of a number of important bacterial pathogens in this region, better informing empiric treatment strategies for travelers to these regions. One of the most common bacterial genera, *Campylobacter*, was identified in over 300 cases of children presenting with diarrhea. Antimicrobial susceptibility testing to *C. jejuni* identified increasing levels of resistance to ciprofloxacin between 2005 and 2008 (21 percent to 65 percent) as well as a moderate level of macrolide resistance among *S. flexneri* and *S. sonnei* isolates (M. Kasper, personal communication). The molecular mechanism of ciprofloxacin resistance in *Campylobacter* spp. was studied using a real-time PCR assay to discriminate between wild-type and mutant alleles that can confer resistance to fluoroquinolones.

## Establishment of an enterics microbiology laboratory—U.S. Army Medical Research Unit, Kenya (USAMRU-K), Kericho, Kenya

A consortium of several regional laboratories conducting infectious disease surveillance and research, USAMRU-K recently moved its principal Enteric Microbiology Laboratory from the Nairobi campus to Kericho, designated the Microbiology Hub in Kericho (or “MHK”). This laboratory includes automated bacterial identification and susceptibility testing, rotavirus enzyme immunoassay (EIA), and limited parasite EIA testing. The MHK has 9,200 square feet of laboratory space with the capacity to process over 100 stool specimens per week and an enhanced molecular epidemiology focus on enteric microorganisms. Ongoing expansion at MHK will provide the capacity to perform important regional epidemiology and clinical trials related to enteric and other bacterial diseases. In addition to supporting clinical microbiology services at Kericho District Hospital, a new protocol was implemented in September 2009 to conduct a case-control study among local residents presenting to Kericho and Kisumu District Hospitals with diarrhea. This project will be expanded with funding from military research programs to support the development of potential field sites for enteric vaccine studies and to conduct observational and clinical trials in military and similar traveler populations throughout the region.

## Establishment of a norovirus reference laboratory to support shipboard and recruit population surveillance—Naval Environmental and Preventive Medicine Unit no. 2 (NEPMU-2), Norfolk, VA

Outbreaks of acute viral gastroenteritis, particularly norovirus, among U.S. military deployed forces, as well as recruit and training populations, are a potential threat to mission capacity and operational readiness, though complete epidemiologic information on the frequency and magnitude of these outbreaks is lacking [5,27-34]. A study by Baily *et al.* reported that outbreaks of acute gastroenteritis were frequent among U.S. and British forces deployed to Iraq and Afghanistan from 2002 to 2007, and of 11 identified outbreaks, 10 had a proven viral cause [35]. Of 84 viral pathogens identified in this series, nearly three-quarters were norovirus. Despite the accumulation of disease threat data, DoD lacks diagnostic capacity under current shore-based and fleet platforms. To remedy this fact, NEPMU-2 is establishing a diagnostic capability for norovirus by developing its laboratory and establishing prospective detection and response activities with recruit training centers, deployed forces and Navy vessels. Collaborative partnerships with the NEPMUs in San Diego and Hawaii have also been developed to enable future surveillance worldwide. Further, the first viral gastroenteritis collection kits containing outbreak guidance, basic epidemiologic collection materials, viral transport media (VTM) supplies, and a thermal shipping box have been deployed for an initial distribution to 30 ships. Data collection for outbreaks during 2010 is anticipated.

## Landscape analysis

### AFHSC-GEIS in the context of global enteric disease surveillance efforts

A growing number of scientific and non-governmental organizations outside the military are showing a growing interest in acute enteric diseases, with several well-organized surveillance activities being established among widespread populations in the developing world (Table 2). These activities include disease-focused networks, such as the WHO-supported regional Rotavirus Surveillance Networks (RSN) [36-38] and the recently established CHOLDInet for cholera and other causes of diarrheal diseases [39]. Under the CDC Global Disease Detection Program, several International Emerging Infections Program (IEIP) sites have been established throughout Africa, Asia and Central America. These programs conduct population-based surveillance to track diseases of global public health importance, including diarrhea.

**Table 2:** Landscape of Current Enteric Disease-Focused Surveillance and Epidemiological Activities

Surveillance System	Lead Institution (s)	Description	Target Populations	Year Established
Rotavirus Surveillance Networks (RSN)	CDC, WHO	Epidemiological support for accelerated rotavirus vaccine introduction. Five networks have been established aligning with WHO regional organizations.	Pediatric populations, global	2000

Cholera and other diarrheal infection network (CHOLDInet)	WHO	Strengthen laboratory capacity for monitoring and rapid detection of cholera and other causes of diarrheal diseases to advance the application of control measures.	Pediatric populations, developing world	2009

International Emerging Infections Program (IEIP)	CDC	Six sites established in Asia (Bangladesh, China, Thailand), Africa (Egypt, Kenya), Central America (Guatemala) with various activities related to enteric surveillance including demographic health surveillance systems and acute diarrhea surveillance.	Adult and pediatric populations, developing world	2001

Global Enteric Multi-Center Surveillance Study (GEMS)	UM-CVD, BMGF	Five-year, multi-center study in Asia (Bangladesh, India, Pakistan), Africa (Gambia, Kenya, Mali, Mozambique) funded by the Bill & Melinda Gates Foundation to quantify the burden and identify the microbiologic etiology of severe diarrheal disease among children 0-59 months of age living in developing nations, for the purpose of addressing limitations of current epidemiology.	Pediatric populations, developing world	2006

Network for the Study of Malnutrition and Enteric Diseases (MAL-ED)	Foundation for NIH, Fogarty International Center, BMGF	Five-year ($30 million), multi-site (eight) project in Africa (South Africa, Tanzania), Asia (Bangladesh, India, Nepal, Pakistan), and South America (Brazil, Peru) with aims to incorporate epidemiology and pathophysiology in a longitudinal study of children from birth to 24 months, to better understand pathogen-related undernutrition and impairment of gut and immune function.	Pediatric populations, developing world	2009

Foodborne Disease Active Surveillance Network (FoodNet)	CDC, USDA, FDA	Multicenter network (10 U.S. sites) with active surveillance for foodborne diseases and related epidemiologic studies designed to help public health officials better understand the epidemiology and burden of foodborne diseases in the United States and disseminate information that can lead to improvements in public health practice.	All ages, U.S.	1995

National Antimicrobial Resistance Monitoring System (NARMS)	FDA, CDC, USDA	Prospective monitoring of the occurrence of antimicrobial resistance of zoonotic pathogens from human diagnostic specimens, retail meats and food animals, many of which are the leading pathogens causing acute enteric illness in the United States.	All ages, U.S.	1996

GeoSentinal Network (GSN)	ISTM, CDC	A multi-site network of 48 globally dispersed medicine clinics on all continents (17 in the United States and 31 in other countries) with aims of worldwide communication and data collection network for the surveillance of travel-related morbidity. The GSN is based on the concept that these clinics are ideally situated to effectively detect geographic and temporal trends in morbidity among travelers, immigrants and refugees.	Adult travelers, global	1995

Military Infectious Diseases Research Program (MIDRP)	DoD	This is a DoD-mandated research program with the purpose of developing effective vaccines and other countermeasures against leading causes of infectious diarrhea in deployed Army, Navy/Marine Corps, and Air Force personnel. This research program includes basic science/discovery efforts, pre-clinical and clinical development, as well as supporting epidemiological studies and clinical trials at the DoD overseas laboratories.	U.S. military and other traveler populations	1970s

Infectious Diseases Clinical Research Program (IDCRP)	DoD	With a mission of conducting research in clinically important infectious disease threats to the warfighter and military community, the IDCRP has established a multi-site travel medicine prospective study that includes an epidemiological study of travelers’ diarrhea and its post-infectious sequelae. The study creates a platform to conduct interventional and diagnostic studies.	U.S. military and beneficiary traveler populations	2006

Two additional research efforts**—**the Global Enteric Multi-Center Surveillance (GEMS) study and the Network for the Study of Malnutrition and Enteric Diseases (MAL-ED)**—**are designed to provide data needed to guide the development and implementation of enteric vaccines and other public health interventions to reduce the morbidity and mortality of diarrheal diseases, as well as to study the relationships between malnutrition and enteric infections.

Beyond the focus on specific diseases and pediatric populations of the developing world, other surveillance activities focus on acute enteric infections within the United States. These include the Foodborne Diseases Active Surveillance Network (FoodNet) [40] and the National Antimicrobial Resistance Monitoring System (NARMS). The GeoSentinel Network (GSN), established in 1995 by the International Society of Travel Medicine (ISTM) and the CDC, is a worldwide communication and data collection network for the study of travel-related morbidity conducted through participating travel clinics worldwide [41]. The DoD Military Infectious Diseases Research Program (MIDRP) has a primary mission of developing vaccine countermeasures to prevent the major infections, including bacterial diarrhea, encountered during deployment. Within this sustained research and development program, surveillance and epidemiological research are long-standing components; MIDRP’s support to the overseas laboratories helps assess the pathogen-specific burden of disease and the establishment of field sites for interventional studies in military and host-national pediatric populations. A number of these efforts are designed for sustainment of activity (RSN, IEIP, MIDRP, FoodNet, NARMS, GSN) while some have only been recently established (CHOLDInet, MAL-ED), and others are likely time-limited (GEMS, MAL-ED). It is within this context that the AFHSC-GEIS mission to strengthen the surveillance and response capabilities of the United States to infectious diseases that threaten to global public health and military readiness should be considered.

## Strengths, weaknesses, opportunities and threats

**Figure 1:**
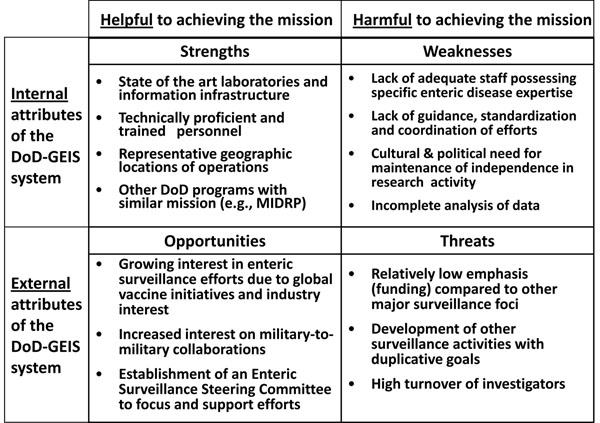
Strength, Weakness, Opportunities and Threats Analysis Matrix for a AFHSC-GEIS Enteric Surveillance Network

The strengths of the current AFHSC-GEIS system include its extensive laboratory infrastructure and technically proficient personnel (Figure 1). Furthermore, the current surveillance activities are leveraged not only by other AFHSC-GEIS initiatives but also by other DoD programs (e.g., MIDRP) with related missions such as field epidemiology and diagnostic test evaluation.

Alongside these strengths, there are also important weaknesses. Whereas some laboratories have principal investigators with special expertise in enteric disease epidemiology, this is not universal across the entire AFHSC-GEIS network. An overall lack of strategic guidance within the enteric diseases program was found in a program review nearly a decade ago [42]. The overseas laboratories are quite diverse and have had considerable latitude for the past decade in study design and the target surveillance populations. This has resulted in varied and successful studies but also in a system with a limited ability to compare data across regions.

This diverse network could be strengthened with an alternative system that uses standardized case definitions, eligibility criteria, basic demographic and clinical data, and advanced pathogen characterization including antimicrobial resistance testing. Such an approach could continue to meet individual laboratory missions while increasing the quality of study design, with the goal of being able to generalize findings across populations and over time. Eventually, the AFHSC-GEIS network could develop into an integrated platform for multi-center studies, with emphases to include novel diagnostic testing and new pathogen discovery.

Other major areas of future interest for AFHSC-GEIS include the link between acute enteric infections and chronic sequelae. More than 50 years ago, the initial descriptions of post-infectious functional bowel disorders were reported [43,44], and two recent systematic reviews have reported that roughly one out of 10 people who develop travelers’ diarrhea will go on to acquire post-infectious irritable bowel syndrome (PI-IBS), despite normal preexisting bowel habits [45,46]. Though PI-IBS may not be as debilitating as some other, less common sequelae of infectious diarrhea such as reactive arthritis [47], the Guillain-Barre syndrome [48] or inflammatory bowel disease [49], the attributable burden of PI-IBS in terms of impact on individual servicemember health, as well as medical readiness, needs consideration similar to other important long-term impacts of combat such as traumatic brain injury and posttraumatic stress disorder. PI-IBS has been described to persist in 57 percent of patients after six years in one study and in 76 percent after five years in another [50,51]. These illnesses decrease the quality of life of those afflicted, and the economic impact is considerable [52]. Although the attributable fraction of IBS caused by domestic foodborne illness is unknown, it is likely large given the frequency of these illnesses [53]. The impact of these infections on deployed servicemembers and on the residents of developing countries, particularly children, remain unclear, and their study may provide AFHSC-GEIS with an important potential avenue of investigation.

While the strengths and weaknesses of the AFHSC-GEIS system will factor in the future direction of enteric surveillance, there are a number of external factors that may also influence the ultimate surveillance strategy. Interest in the area of enteric diseases is growing, with new private industry and philanthropic interest in developing vaccines for military and civilian populations (including diarrhea vaccines). The DoD has placed a higher-level emphasis on conducting more military-to-military engagements. If appropriately aligned, these trends could represent new opportunities and garner additional external support.

Currently, the DoD funding allocated to enteric disease surveillance activities is relatively lower than that allocated to other AFHSC-GEIS disease pillars. Given the real and ever-present impact of diarrheal diseases experienced by deployed military troops, and the appearance of additional burden associated with chronic disease sequelae of these acute infections, an effort to re-emphasize the critical importance of understanding and preventing these infections is needed. In recent years, the enteric disease research and epidemiological landscape has changed significantly with the establishment of GEMS, MAL-ED, and CDC IEIP sites as previously mentioned. These multinational well-funded initiatives may be seen as overlapping with many AFHSC-GEIS activities. A strategic assessment is needed to determine the future goals of AFHSC-GEIS, given limited resources and a potentially constrained fiscal environment.

## Future vision

The knowledge gained through studies of deployed military populations has directly contributed to our understanding of acute enteric infections and their burden among military personnel and families stationed in high-risk areas overseas [9,54,55]. To meet the needs and mission of the DoD and the global public health community, many of the traditional beliefs about the preferred types of surveillance activities may need re-evaluation. For example, the value of year-to-year tracking of antimicrobial drug resistance among enteric pathogens needs to be assessed. While it is true that resistance to enteropathogens may complicate the therapy of diarrhea, most of these infections currently go untreated and generally are self-limited in nature [56]. A surveillance strategy is needed that considers the scope, frequency of assessment, target populations and regions of interest.

While surveillance among U.S. military and adult traveler populations may be considered ideally aligned with the AFHSC-GEIS mission, there are challenges in consistent availability of these populations. Often it is attractive to perform regional surveillance among local populations as a surrogate for understanding disease burden and risks; however, the data collected from these efforts, specifically related to enteric diseases, are not able to be generalized to deployed U.S. military or adult traveler populations in the region. Secondary benefits in conducting studies among local populations include capacity building and improved host nation relations. While children in developing countries may serve as good surrogates in terms of immune naïveté, there may be important differences in pathogen exposure, environment, host-response and risk behavior that may impact the direct generalization of risk to U.S. military populations. For example, among studies conducted by NAMRU-3 in deployed military personnel participating in Operation Bright Star exercises in Egypt, the common enterotoxigenic *E. coli* toxin phenotypes were heat-labile toxin (LT) 17 percent, heat-stable toxin (ST) 56 percent, and LTST 26 percent; and predominant colonization factor prevalences were CS6 (32 percent), CS3 (8 percent), and CFA/1 (5 percent) [16-18,22,57]. This is in contrast to studies among Egyptian pediatric populations less than five years of age in Egypt, where the toxin prevalence (LT 36 percent; ST 51 percent; LTST 12 percent) and colonization factor profiles (CS6 9 percent; CS3 0.6 percent; CFA/1 8 percent) were quite different [10,58-61]. The implications of the varied prevalence of ETEC strain phenotypes in two different populations with disease from the same region is critically important, not only in design considerations of current ETEC vaccines under development by the DoD, but also in the selection of appropriate surveillance populations. Furthermore, while host nation military personnel may seem to be a surrogate target population, the acceptability of surveillance in these populations is complicated by their higher probability of acquired immunity to many pathogens, an often-high prevalence of chronic parasite carriage, and potential sensitivities in collaborating with an institution that may not have a broadly favorable reputation within the country. Nonetheless, given limited DoD funding and resources, the value of which target populations are best matched for which objective under an AFHSC-GEIS mission needs further consideration.

The leadership at AFHSC-GEIS has recognized the importance of this assessment and the need for refinement of this program and has established the charter of an Enteric Surveillance Steering Committee (ESSC) to guide the current laboratory network into the future. The development of a true network—implying coordination and standardization in a broader context—would add significantly to the value and achievement of the AFHSC-GEIS mission. However, a cautious approach is needed because each laboratory has variable expertise, available populations and interests. Strategies to achieve a coordinated network could range from providing guidance on laboratory methodology to a common core data element which could be collected on all case patients enrolled in the various studies (e.g., similar to what is done with FoodNet reporting sites) to run global and longitudinal analyses. While comprehensive standardized protocols (e.g., similar to RSN) might be difficult to implement in today’s landscape, certain aspects including microbiological methods, antimicrobial resistance testing and clinical data capture could be harmonized across surveillance protocols at each of the sites. The relatively low funding for enteric disease surveillance compared to other disease pillars needs to be addressed. Given the real and ever-present impact of diarrheal diseases experienced by local populations and deployed military troops, the critical importance of enteric infections needs to be re-emphasized. This importance, as well as identified gaps that can be filled, should be met with dedicated programmatic funding allocated to enteric research.

## Conclusion

A review of AFHSC-GEIS global enteric surveillance efforts for the most recent fiscal year (2009) shows a diverse network needing refinements in vision and standardization, and a strategically directed surveillance effort. Recent changes in the global enteric disease surveillance and research landscape provide the necessary impetus. The strengths of the DoD laboratory network and individual investigators have generated a number of positive contributions including: longitudinal data on emerging antimicrobial resistance; expansive surveillance activities in multiple countries in practically all regions of the globe; the important threat information been obtained from direct study of deployed populations in Iraq and Afghanistan; the capability of supporting outbreak response throughout the world in both military and non-military populations; and the intangible benefits through medical diplomacy. The challenges in executing networked studies with operational significance in U.S. military populations include initiating multi-center surveillance protocols in new host countries with varying regulatory requirements, and the shifting allocation of resources and emphasis away from enteric diseases to other concurrent public health treats (such as with avian influenza/pandemic influenza). However, these challenges are countered by the benefit of achieving a true global network of laboratories aligned in mission and outcome in mitigating the enteric disease threat.

Future efforts in enteric disease surveillance by AFHSC-GEIS will be shaped by its newly established ESSC. As recommended in a prior AFHSC-GEIS program review [42] and explored in this landscape analysis, areas for direction include: (1) provision of specific guidance to laboratories regarding the goals of AFHSC-GEIS and the qualities that AFHSC-GEIS projects are expected to possess, in addition to active assistance of laboratories in developing project plans and periodic scientific guidance for projects under way; (2) a mechanism for structured project review that permits adequate time for project conduct between reviews, results in timely feedback to investigators and is carried out by a diverse panel of experts; (3) consistent interaction with staff directing AFHSC-GEIS projects to monitor project progress, potential for collaboration and needs for assistance; and (4) an improved means of collecting and distributing surveillance data and other information in a timely manner.

These recommendations will guide the focus of the newly chartered ESSC as its members identify strategic issues, develop new goals, create implementation plans and evaluate future progress. The task that lies ahead for the ESSC is to answer the questions of “What do we do?”, “For whom do we do it?”, and “How can we do it well?” The enhanced coordination and improved scientific review possibilities of the ESSC will help AFHSC-GEIS build upon its past successes in enteric disease surveillance, continuing to take advantage of the DoD laboratories’ history, experience and global reach to answer these questions and achieve the AFHSC-GEIS mission.

## Competing interests

The authors declare that they have no competing interests.
